# Acquisition of Cell–Cell Fusion Activity by Amino Acid Substitutions in Spike Protein Determines the Infectivity of a Coronavirus in Cultured Cells

**DOI:** 10.1371/journal.pone.0006130

**Published:** 2009-07-02

**Authors:** Yoshiyuki Yamada, Xiao Bo Liu, Shou Guo Fang, Felicia P. L. Tay, Ding Xiang Liu

**Affiliations:** 1 Institute of Molecular and Cell Biology, Proteos, Singapore, Singapore; 2 School of Biological Sciences, Nanyang Technological University, Singapore, Singapore; University of Minnesota, United States of America

## Abstract

Coronavirus host and cell specificities are determined by specific interactions between the viral spike (S) protein and host cell receptor(s). Avian coronavirus infectious bronchitis (IBV) has been adapted to embryonated chicken eggs, primary chicken kidney (CK) cells, monkey kidney cell line Vero, and other human and animal cells. Here we report that acquisition of the cell–cell fusion activity by amino acid mutations in the S protein determines the infectivity of IBV in cultured cells. Expression of S protein derived from Vero- and CK-adapted strains showed efficient induction of membrane fusion. However, expression of S protein cloned from the third passage of IBV in chicken embryo (EP3) did not show apparent syncytia formation. By construction of chimeric S constructs and site-directed mutagenesis, a point mutation (L857-F) at amino acid position 857 in the heptad repeat 1 region of S protein was shown to be responsible for its acquisition of the cell–cell fusion activity. Furthermore, a G405-D point mutation in the S1 domain, which was acquired during further propagation of Vero-adapted IBV in Vero cells, could enhance the cell–cell fusion activity of the protein. Re-introduction of L857 back to the S gene of Vero-adapted IBV allowed recovery of variants that contain the introduced L857. However, compensatory mutations in S1 and some distant regions of S2 were required for restoration of the cell–cell fusion activity of S protein carrying L857 and for the infectivity of the recovered variants in cultured cells. This study demonstrates that acquisition of the cell–cell fusion activity in S protein determines the selection and/or adaptation of a coronavirus from chicken embryo to cultured cells of human and animal origins.

## Introduction

Interspecies adaptation, replication and transmission in cells are essential steps for an animal virus to emerge successfully in a human population. Virus-cell and cell–cell membrane fusion, mediated by fusion proteins associated with viral envelope, is crucial for the entry of enveloped viruses into cells and for rapid spread of infection to the neighboring cells. This membrane fusion process may, therefore, be a limiting point for efficient adaptation and infection of an animal virus in cells from a different host species. In this study, we report that acquisition of the cell–cell fusion activity by point mutations in the spike (S) protein of avian coronavirus infectious bronchitis virus (IBV) plays a critical role in adaptation and/or selection of a variant that infects cultured cells.

Coronavirus is a large family of enveloped, positive-stranded RNA viruses that cause respiratory and intestinal infections in avian and mammalian species [1]. IBV, the prototype member of coronavirus, causes highly contagious diseases in chicken and is a constant threat to the poultry industry. Coronavirus was traditionally considered to have narrow host specificities [2]. However, the outbreaks of severe acute respiratory syndrome (SARS), a serious zoonotic transmission event caused by a novel coronavirus, demonstrate that a certain coronvirus species may exhibit wider host specificities and suggests the possibility of cross-species transmission of animal coronaviruses to human [3], [4]. Cross-species transmission was also observed in coronavirus transmissible gastroenteritis virus (TGEV) and human coronavirus OC43 [5]–[7]. These events highlight the importance of understanding the mechanisms of interspecies adaptation and transmission of coronavirus.

The Beaudette strain of IBV was previously adapted to embryonated chicken eggs. This embryo-adapted IBV strain was subsequently adapted to cultured cells originated from chicken and monkey. For example, the virus was adapted by serial passages to primary chicken kidney (CK) cells [8], [9] and the African green monkey kidney cell line Vero cell [8]–[13]. Furthermore, the Vero-adapted IBV is able to infect cultured human and animal cell lines [14], [15].

In a previous report, a total of 49 amino acid substitutions was found during adaptation of IBV from chicken embryo (EP3) to Vero cells (p65) [10]–[12]. Among them, 26 amino acid substitutions are in the S protein [10]. In this study, expression of S protein cloned from IBV strains EP3, CK, passage 7 (p7) and p65 of Vero-adapted IBV showed induction of cell–cell fusion by S(CK), S(p7) and S(p65) constructs. However, no formation of syncytial cells was observed in cells expressing S(EP3). Construction of chimeric S constructs and site-directed mutagenesis studies identified a leucine to phenylalanine substitution at the amino acid position 857 in the heptad repeat 1 region (L857-F) that confers the non-fusogenic S protein to fusogenic. Re-introduction of the F857-L mutation back to the genome of Vero-adapted IBV (p65) showed rescue of virus containing the F857-L mutation. However, compensatory mutations occurred in the S1 region that could rescue the cell–cell fusion activity of S constructs carrying the F857-L mutation.

## Materials and Methods

### Cells and viruses

Cells were maintained in DMEM supplemented with 10% newborn calf serum. The Vero-adapted IBV and recombinant vaccinia/T7 virus was propagated and titrated on Vero cells. Virus stocks were kept at −80°C until use.

### Immunofluorescent staining

Cell monolayers grown on 4 well slide chambers were infected with vaccinia/T7 virus for 1 hour followed by transfection of indicated plasmids using the Effectene transfection kit (Qiagen). At 12 hours post-transfection, cells were washed with phosphate buffered saline (PBS) supplemented with 10% normal goat serum, fixed with 4% paraformaldehyde in PBS for 15 minutes, and permeabilized with 0.2% Triton X-100. Immunofluorescent staining was performed by incubation of cells with rabbit anti-IBV S polyclonal antibodies and subsequently with FITC-conjugated anti-rabbit IgG. Cells were examined by fluorescent microscopy and digital images were collected.

### Western blotting

Protein samples were prepared from cells harvested at 12 hours post-transfection, separated by SDS-PAGE and transferred to PVDF membranes. The membranes were incubated with rabbit anti-IBV S polyclonal antibodies or mouse anti-β-tubulin monoclonal antibody (Sigma Aldrich), and subsequently with HRP-conjugated anti-rabbit or -mouse IgG (DAKO). Polypeptides were detected using the enhanced chemiluminescence (ECL) detection reagents (Amersham).

### Flow cytometry

Vero cells were transfected as described above, and harvested at 12 hours post transfection. Cells were washed once with PBS, resuspended in blocking buffer containing 20% FBS and 1% BSA in PBS, and incubated on ice for 30 minutes. Subsequently, cells were incubated with 0.1% saponin in FACS washing buffer containing 2.5% FBS and 0.05% sodium azide in PBS for 10 minutes at room temperature when required. Immunofluorescent staining was carried out with 1∶100 diluted rabbit anti-IBV S polyclonal antibodies, and 1∶20 diluted FITC-conjugated swine anti-rabbit antibody (DAKO). After washing two times with the FACS washing buffer, cells were fixed with 1% ice cold paraformaldehyde and analyzed by flow cytometry.

### RT-PCR and Sequencing

Viral RNA was extracted from the culture supernatants or infected cells using the RNeasy Mini Kit (Qiagen) according to the manufacturer's instructions. RT-PCR was performed using the Expand Reverse Transcription and High Fidelity PCR Kits (Roche). The PCR products were cloned into PCR®-XL-TOPO® vector (Invitrogen) and sequenced by automated sequencing.

### Construction of full-length wild type and mutant IBV cDNAs, in vitro transcription and electroporation

Construction of the full-length IBV cDNA clones from p65 of Vero-adapted IBV was previously reported [16], [17]. The F857-L point mutation was introduced into the corresponding fragment using QuickChange site-directed mutagenesis kit (Stratagene), and subsequently ligated into the full-length cDNA clone.

The full-length transcripts were generated in vitro using the mMessage mMachine T7 kit (Ambion) according to the manufacturer's instructions with certain modifications. Briefly, 30 µl of transcription reaction with a 1∶1 ratio of GTP to cap analog were sequentially incubated at 40.5°C for 25 minutes, 37.5°C for 50 minutes; 40.5°C for 25 minutes, and 37.5°C for 20 minutes. The transcripts were extracted with phenol/chloroform.

Vero cells were grown to 90% confluence, trypsinized, washed twice with cold PBS, and resuspended in PBS. RNA transcripts were added to 400 µl of Vero cell suspension in an electroporation cuvette, and one electrical pulse at 450 V, 50 µF was given using a Bio-Rad Gene Pulser II electroporator. The transfected Vero cells were cultured overnight in 1% FBS-containing MEM in a 60 mm dish or a six-well plate and further cultured in MEM without FBS.

### Construction of plasmids

The S genes from different passages of the Vero-adapted IBV strain and CK-adapted IBV were amplified and cloned into pKT0 vector [18]. Chimeric S constructs were made by overlapping PCR [19]. Point mutations were introduced by site-directed mutagenesis using the Quikchange™ kit (Stratagene). All constructs were confirmed by automated nucleotide sequencing.

## Results

### Cell-cell fusion activity of S proteins cloned from EP3 and CK-adapted Beaudette strain of IBV

**Figure 1:**
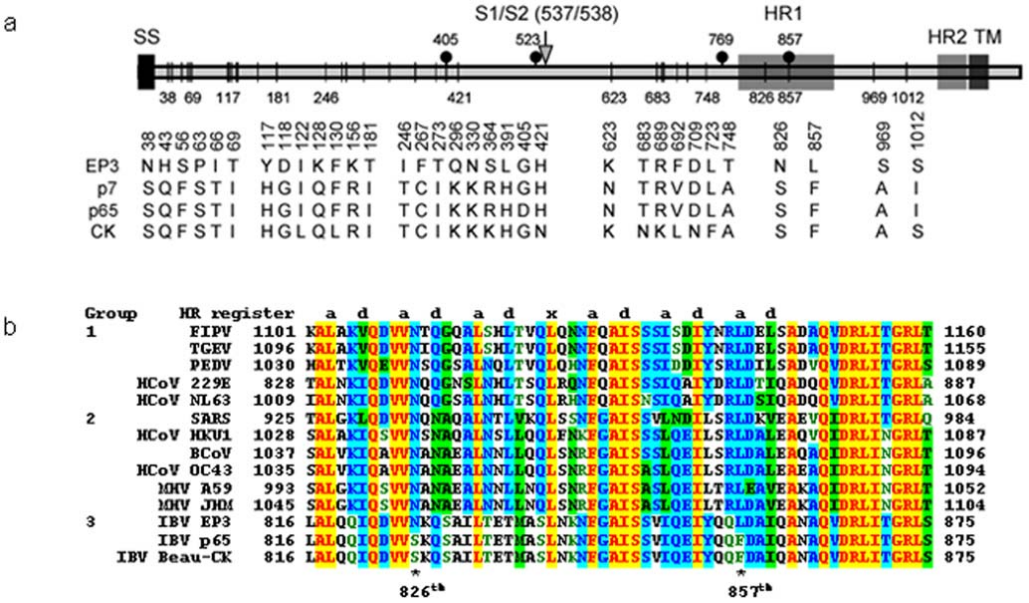
Summary of amino acid substitutions in the IBV S genes from chicken embryo- and cell culture-adapted IBV Beaudette strains. a. Schematic diagram of the IBV S gene structure and functional domains. Also shown are amino acid substitutions in the S protein derived from IBV strains EP3 (accession No. AAY24433), p7 (accession No. AAY21245), p65 (accession No. AAY24433) and CK (accession No. CAC39114). Black dots indicate the identified amino acid positions that affect the cell–cell fusion activity of S protein in this study. b. Comparison of amino acid sequences in the heptad repeat I region of S proteins from coronavirus feline infectious peritonitis virus (FIPV), transmissible gastroenteritis virus (TGEV), porcine epidemic diarrhea virus (PEDV), human coronavirus 229E (HCoV 229E), human coronavirus NL63 (HCoV NL63), SARS-CoV, human coronavirus HKU1 (HCoV HKU1), bovine coronavirus (BCoV), mouse hepatitis virus (MHV) A59, MHV JHM, IBV strains EP3, p65 and Beau-CK.

**Figure 2:**
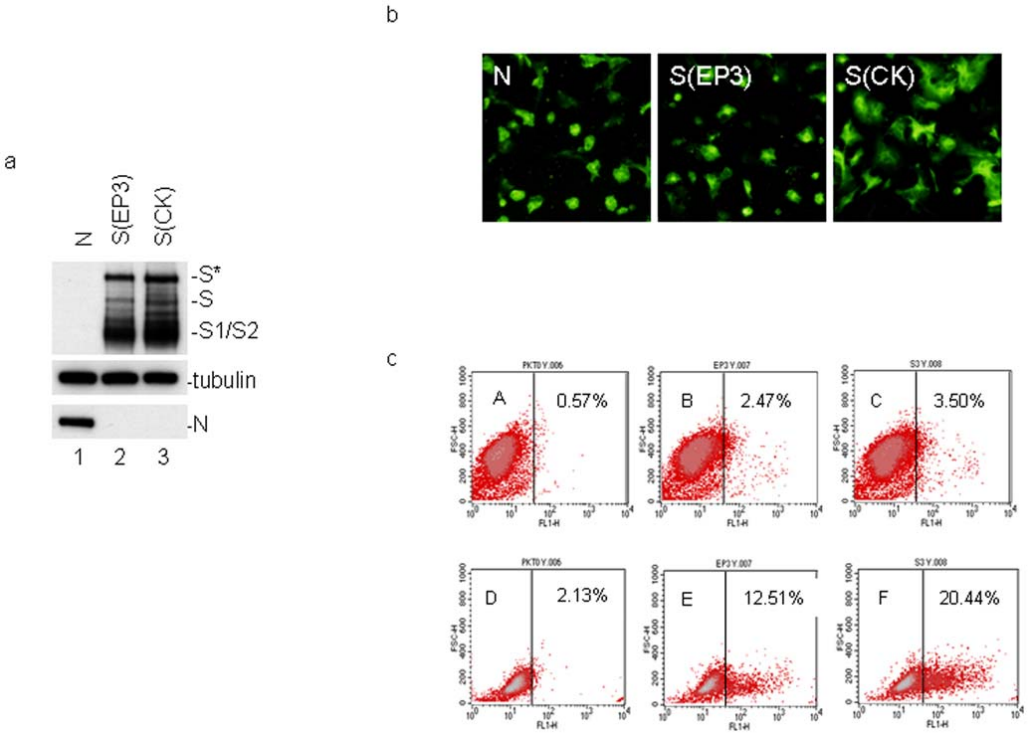
Induction of cell–cell fusion by S(EP3) and S(CK) constructs. a. Western blot analysis of cells expressing IBV N (lane 1), S(EP3) (lane 2) and S(CK) (lane 3). Vero cells were infected with vaccinia/T7 recombinant virus and transfected with the indicated constructs. Cells were harvested at 12 hours post-transfection and lysates prepared. The viral protein expression was analyzed by Western blot with rabbit anti-IBV S and anti-IBV-N polyclonal antibodies. The same membrane was also probed with anti-β-tubulin monoclonal antibody as a loading control. b. Detection of cell–cell fusion by indirect immunofluorescence. Vero cells were infected with vaccinia/T7 recombinant virus and transfected with the indicated constructs. At 12 hours post-transfecion, cells were fixed with 4% paraformaldehyde and stained with rabbit anti-IBV S and anti-IBV N polyclonal antibodies. c. Cell surface expression of S(EP3) and S(CK) constructs. Vero cells were infected with the vaccinia/T7 recombinant virus and transfected with the indicated constructs. At 12 hours post-transfecion, cells were either stained directly with 1∶100 diluted rabbit anti-IBV S polyclonal antibodies (panels A, B and C), or permeabilized with 0.1% saporin following by staining with the same antibodies (panels D, E and F). The cells were then incubated with 1∶20 diluted FITC-conjugated swine anti-rabbit antibody, fixed with 1% ice cold paraformaldehyde and analyzed by flow cytometry.

Acquisition of the cell–cell fusion activity is essential for selection and adaptation of coronavirus IBV from chicken embryo to cultured cells [10]. Sequence comparison of two S protein constructs, S(EP3) and S(CK), cloned from EP3 and CK-adapted IBV strains, respectively, showed amino acid substitutions at 31 positions (Fig. 1a). The cell–cell fusion activity of these two S constructs was analyzed by transfection into Vero cells using the vaccinia/T7 recombinant virus system. Western blot analysis showed the presence of major forms of S protein, including the 180-kDa glycosylated (S*) and 130-kDa unglycosylated full-length S (S) and the cleaved S1 and S2 species (S1/S2) in cells expressing the two constructs (Fig. 2a, lanes 2 and 3). It was noted that the expression level of S(CK) was higher than S(EP3) (Fig. 2a). As a negative control, cells transfected with IBV N protein were included, and the expressed N protein was detected by Western blot with anti-N antibodies (Fig. 2a, lane 1). Immunofluorescent staining of Vero cells expressing S(CK) clearly showed syncytia formation at 12 hours post-transfection (Fig. 2b, panel S(CK)). However, in Vero cells expressing S(EP3), no obvious syncytia was observed (Fig. 2b, panel S(EP3)). In the negative control cells, no fusion of the transfected cells was detected (Fig. 2b, panel N).

To investigate the possibility that intrinsic differences in cell surface translocation of the two S constructs may affect their cell–cell fusion activity, cell surface expression of the two proteins was analysed by flow cytometry after immunofluorescent staining with anti-S antiserum. As shown in Fig. 2c, 0.57% of nonpermeabilized (panel A) and 2.13% of permeabilized (panel D) cells expressing empty plasmid exhibited background staining. Under nonpermeabilizing conditions, 1.9% (2.47–0.57) cells expressing S(EP3) (panel B) and 2.93% (3.5–0.57) cells expressing S(CK) showed positive staining. After permeabilizing with 0.1% saponin, 10.38% (12.51–2.13) of cells expressing S(EP3) protein (panel E) and 18.31% (20.44–2.13) of cells expressing S(CK) (panel F) showed positive staining. These results confirm that the two S proteins could be translocated to the cell surface with a similar efficiency.

### Acquisition of the cell–cell fusion activity by mutation of a conserved leucine residue to phenylalanine (L857-F) in the heptad repeat 1 region of S protein

**Figure 3:**
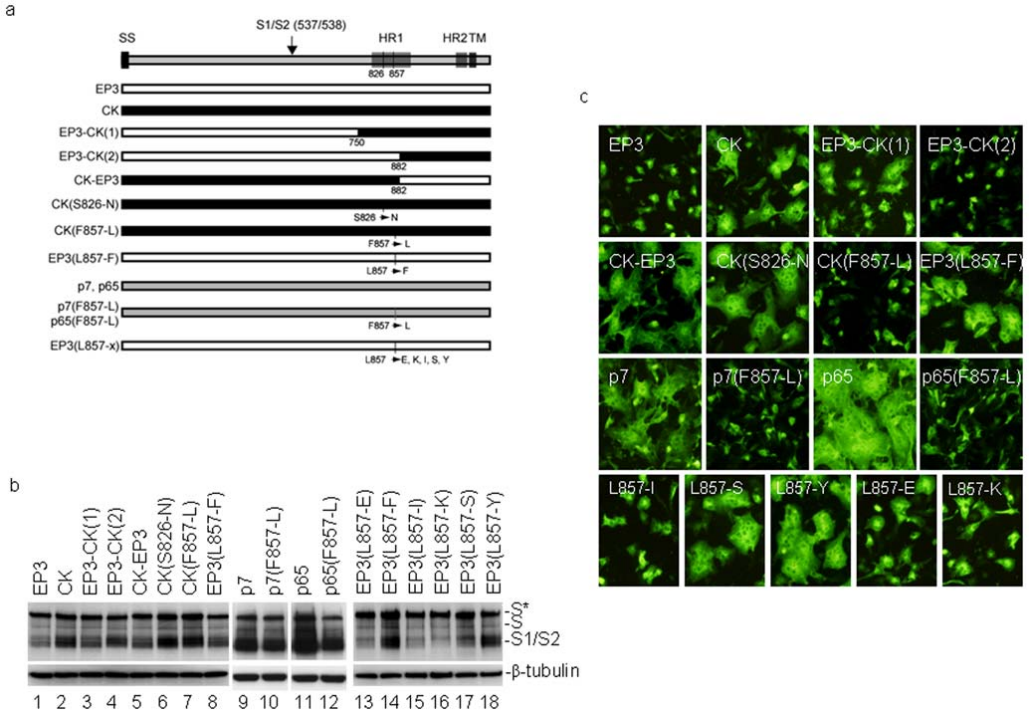
Acquisition of the cell–cell fusion activity by L857-F mutation in the heptad repeat 1 region and mutational analysis of the L857 residue. a. Schematic diagram of various wild type and mutant S constructs as well as several chimeric constructs used in this study. b. Western blot analysis of cells expressing wild type, mutants, and chimeric IBV S constructs. Vero cells were infected with vaccinia/T7 recombinant virus and transfected with the indicated constructs. Cells were harvested at 12 hours post-transfection and lysates prepared. The viral protein expression was analyzed by Western blot with rabbit anti-IBV S antibodies. The same membrane was also probed with anti-β-tubulin monoclonal antibody as a loading control. c. Detection of cell–cell fusion by indirect immunofluorescence. Vero cells were infected with vaccinia/T7 recombinant virus and transfected with the indicated S constructs. At 12 hours post-transfecion, cells were fixed with 4% paraformaldehyde and stained with rabbit anti-IBV S polyclonal antibodies.

To map the amino acid mutation(s) responsible for acquisition of the cell–cell fusion activity of S(CK), three chimeric constructs were first made. Construct EP3-CK(1) was made by replacing the C-terminal 412 amino acid region of S(EP3) with the corresponding region from S(CK), EP3-CK(2) was made by replacing the C-terminal 280 amino acid region of S(EP3) with the corresponding region from S(CK), and CK-EP3 was made by replacing the N-terminal 882 amino acid region of S(EP3) with the corresponding region from S(CK) (Fig. 3a). Western blot analysis of cells expressing these constructs detected the S1 and S2 species as well as the glycosylated and unglycosylated forms of the full-length S protein (Fig. 3b, lanes 3–5). Immunofluorescent staining showed cell–cell fusion and syncytia formation in cells expressing both EP3-CK(1) and CK-EP3 (Fig. 3c, panels EP3-CK(1) and CK-EP3), but not EP3-CK(2) (Fig. 3c, panel EP3-CK(2)). The relative cell–cell fusion activities of these S constructs were semi-quantitatively defined by comparing the average size of syncytia induced by different S constructs with the average size (considered as 1) of cells expressing S(EP3), and are listed in the order from high to low as follows: CK-EP3>CK = EP3-CK(1)≫EP3 = EP3-CK(2) (>indicates the relative activity is within 1 fold higher, and ≫indicates more than 1 fold higher). These results demonstrate that the region between amino acids 750 and 882 may determine the fusogenic difference between S(EP3) and S(CK).

Examination of this region showed two amino acid substitutions from S(EP3) to S(CK), *i.e.* N826 to S and L857 to F (Fig. 1b). To determine which amino acid substitution dictates the fusogenic change, three mutant constructs were made. Constructs CK3(S826-N) and CK(F857-L) were made by mutation of the S826 and F857 residues in S(CK) to N and L, respectively (Fig. 3a). Construct EP3(L857-F) was made by mutation of the L857 residue in S(EP3) to F (Fig. 3a). Western blot analysis of cells expressing these constructs detected the S1 and S2 species as well as the full-length forms (Fig. 3b, lanes 6–8). Immunofluorescent staining showed formation of syncytia in cells expressing CK(S826-N) (Fig. 3c, panel CK(S826-N), suggesting that mutation of S836 to N did not affect the cell–cell activity of S(CK). Cell-cell fusion and syncytia formation were also observed in cells expressing EP3(L857-F) but not CK(F857-L) (Fig. 3c, panels EP3(L857-F) and CK(F857-L)), demonstrating that the L857-F mutation introduced into S(EP3) renders the protein fusogenic in cultured cells. On the other hand, mutation of the F857 residue to L totally abolishes the fusion activity of S(CK) (Fig. 3c, panel CK(F867-L). The relative cell–cell fusion activities of these S constructs are CK(S826-N)>EP3(L857-F)≫EP3 = CK(F857-L). These results confirm that S(CK) gains the cell–cell fusion activity by L857-F mutation in the heptad repeat 1 region of the protein.

**Figure 4:**
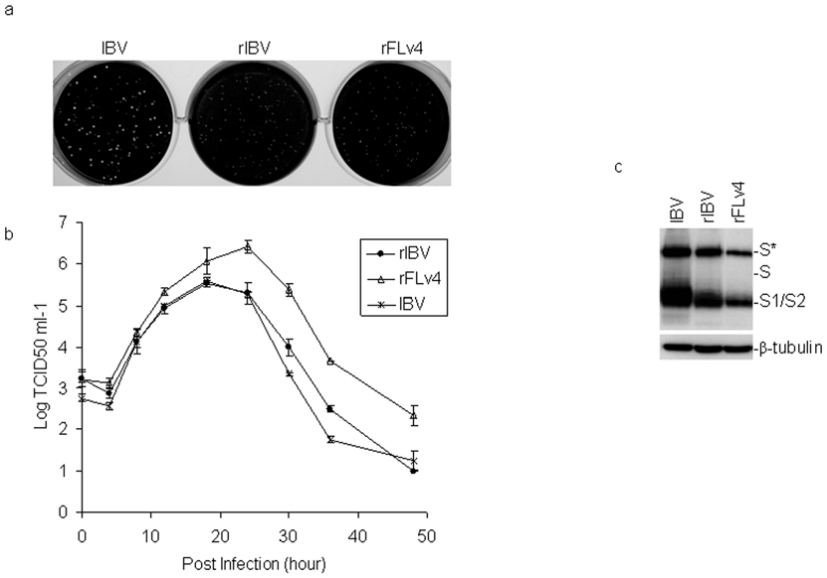
Analysis of the effect of L857 residue on IBV infectivity and growth properties by re-introduction of F857-L back into the genome of Vero-adapted IBV p65. a. Comparison of the plaque sizes of wild type IBV (IBV), wild type recombinant IBV (rIBV) and variant 4 (rFLv4). Confluent monolayers of Vero cells grown on 6-well plates were infected with wild type IBV (IBV), wild type recombinant IBV (rIBV) and variant 4 (rFLv4), and incubated in the presence of 0.5% carboxymethy cellulose. At 2 days post-infection, cells were washed, fixed with 4% formaldehyde, and stained with 0.1% toluidine blue. b. Comparison of the growth curves of wild type IBV (IBV), wild type recombinant IBV (rIBV) and variant 4 (rFLv4). Vero cells were infected with viruses, harvested at 0, 4, 8, 12, 18, 24, 30, 36 and 48 hours post-infection, and TCID50 was determined. c. Analysis of S protein expression in cells infected with wild type IBV (IBV), wild type recombinant IBV (rIBV) and variant 4 (rFLv4). Vero cells infected with the indicated viruses with the same MOI were harvested at 16 hours post-infection. The viral protein expression was analyzed by Western blot with rabbit anti-IBV S antibodies. The same membrane was also probed with anti-β-tubulin monoclonal antibody as a loading control.

The F857-L substitution was then introduced into the S constructs cloned from Vero-adapted IBV (p7) and (p65), respectively, generating S(p7) and S(p65) (Fig. 3a). Expression of these constructs showed cell–cell fusion and syncytia formation in cell expressing wild type S(p7) and S(p65), but not the mutant S proteins (Fig. 3c, panel p7, p7(F857-L), p65 and p65(F857-L)). The expression levels of both F857-L mutants were lower than the wild type constructs, but no significant difference in the S1/S2 cleavage was observed (Fig. 3b, lane 9–12). The relative cell–cell fusion activities of these S constructs are p65>p7≫EP3 = p7(F857-L) = p65(F857-L). These data indicate that S protein acquires its cell–cell fusion activity by the L857-F mutation during adaptation to both CK and Vero cells.

Further mutations of the L857 residue to other amino acids based on S(EP3) were made. As shown in Fig. 3a, the L857 was mutated to Y, S, E, I and K, respectively. Expression of these mutant constructs showed that mutations of L857 to Y and S exhibited similar effect on cell–cell fusion as the L857-F mutant. Cell-cell fusion and syncytia formation were observed in cells expressing these two mutants (Fig. 3c). However, much less cell–cell fusion and smaller-sized syncytia were observed in cells expressing L857-E, L857-K and L857-I mutant constructs (Fig. 3c). The relative cell–cell fusion activities of these S constructs are EP3(L857-Y)>EP3(L857-S)>EP3(L857-E)>EP3(L857-I) = EP3(L857-K)>EP3.

### Introduction of the F857-L substitution back to the genome of Vero-adapted IBV and analysis of its effect on viral infectivity in cultured cells

The F857-L mutation was then introduced back to the genome of Vero-adapted IBV by using an infectious clone system based on p65 [16], [17] to test its influence on viral recovery and infectivity. In vitro synthesized full-length transcripts derived from wild type (rIBV) and mutant (FL) clones were introduced into Vero cells by electroporation. At 3 days post-electroporation, syncytia formation was clearly observed in cells electroporated with wild type transcripts. No apparent CPE was observed in cells electroporated with the mutant transcripts at this time point. Upon extension of the incubation time to 6 days, smaller-sized syncytia appeared.

The recombinant wild type and mutant viruses (p0) were recovered from the culture media at 3 and 6 days post-electroporation, respectively, and further propagated on Vero cells for 5 passages. Total RNA was extracted from the culture media of cells infected with each passage of the mutant virus and RT-PCR was carried out to amplify the S gene. The RT-PCR products were cloned, 10 bacterial clones were randomly chosen from p0, and the complete nucleotide sequence of the S gene was determined to confirm if the recovered virus maintains the F857-L substitution. As shown in table 1, L857 was found in all 10 clones. However, only five clones had an identical sequence with the original mutant S gene (type FL), and additional mutations at other positions were found in the other five clones (Table 1). Among them, two clones contain a T773-S substitution (FLv1), one contains an I769-V substitution (FLv2), and two contain Q523-L and I769-V substitutions (FLv3) (Table 1). These results demonstrate that the recovered FL mutant virus from p0 contains a mixed population of quasispecies.

**Table 1:** Summary of amino acid substitutions in S protein from the rescued IBV variants.

Passage	S gene type	No. of clones (No./total)	Amino acid residue					Plaque purification (No./total)
			327	523	769	773	857	
p0	FL	5/10	P	Q	I	T	L	ND
	FLv1	2/10	P	Q	I	S	L	ND
	FLv2	1/10	P	Q	V	T	L	ND
	FLv3	2/10	P	L	V	T	L	ND
p1	FLv3	4/4	P	L	V	T	L	ND
p3	FLv3	4/10	P	L	V	T	L	0/10
	FLv4	6/10	S	L	V	T	L	10/10
p5	FLv3	2/10	P	L	V	T	L	0/10
	FLv4	8/10	S	L	V	T	L	10/10

To investigate which quasispecies would become dominant in the subsequent passages, sequencing analysis of bacterial clones containing the PCR fragments from p1, p3 and p5 was performed. In the four clones chosen from p1, a homogenous S gene with both Q523-L and I769-V (FLv3) mutations was found (Table 1). Subsequent sequencing of clones derived from p3 and p5 each showed that six out of 10 clones from p3 and two out of 10 clones from p5 are FLv3 (Table 1). The dominant clones contain an additional proline to serine substitution at amino acid position 327 (FLv4) (Table 1).

The recovered viruses were then plaque-purified. Compared to wild type IBV, rIBV showed similar growth kinetics in Vero cells (Fig. 4b), but formed slighlty smaller plaques (Fig. 4a) with lower expression level of S protein (Fig. 4c). A total of 20 mutant viruses was plaque-purified from passages 3 and 5, and the S gene of all purified viruses was shown to share the same sequence as FLv4 (Table 1). The FLv4 mutant virus formed similar-sized plaques as rIBV (Fig. 4a) with slightly lower expression of S protein (Fig. 4c). Interestingly, the mutant virus produced up to 10-fold higher titers of virus, compared to rIBV (Fig. 4b).

### Restoration of the cell–cell fusion activity of S(p65) protein carrying the F875-L mutation by compensatory mutations in the S1 region

**Figure 5:**
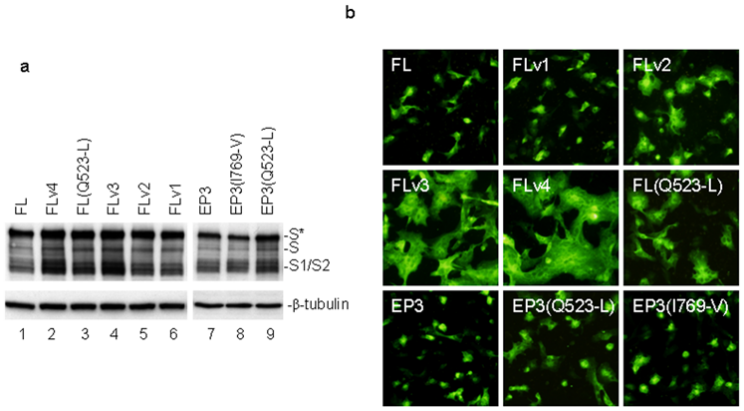
Induction of cell–cell fusion by various L857-containing S constructs. a. Western blot analysis of cells expressing L857-containign S constructs, S(FL), S(FLv1), S(FLv2), S(FLv3) and S(FLv4). Vero cells were infected with vaccinia/T7 recombinant virus and transfected with the indicated constructs. Cells were harvested at 12 hours post-transfection and lysates prepared. The viral protein expression was analyzed by Western blot with rabbit anti-IBV S antibodies. The same membrane was also probed with anti-β-tubulin monoclonal antibody as a loading control. b. Detection of cell–cell fusion by indirect immunofluorescence. Vero cells were infected with vaccinia/T7 recombinant virus and transfected with the indicated S constructs. At 12 hours post-transfecion, cells were fixed with 4% paraformaldehyde and stained with rabbit anti-IBV S polyclonal antibodies.

The cell–cell fusion activity of S proteins cloned from the mutant IBV construct FL and the four variants (FLv1, FLv2, FLv3 and FLv4) was analyzed by expression in Vero cells. Once again, expression of these constructs led to the detection of S1 and S2 species as well as the full-length forms (Fig. 5a). Higher levels of S protein were detected in cells expressing S(FLv3) and S(FLv4), comparing to cells expressing the other two S constructs (Fig. 5a). Immunofluorescent staining showed the formation of giant syncytia in cells expressing S(FLv3) and S(FLv4) (Fig. 5b, panels S(FLv3) and S(FLv4)), but much smaller syncytia were observed in cells expressing S(FLv2) (Fig. 5b, panel S(FLv2)). No obvious cell–cell fusion was observed in cells expressing S(FL) and S(FLv1) (Fig. 5b, panels S(FL) and S(FLv1)). The relative cell–cell fusion activities of these S constructs are FLv4>FLv3>FLv2≫EP3 = FL = FLv1. These results confirm that acquisition of the cell–cell fusion activity is an important step for adaptation of IBV in cultured cells.

Since amino acid difference between S(FLv2) and S(FLv3) was only at the 523^th^ residue, the S(FL(Q523-L)) construct was also created and expressed. The results showed that it displayed a similar cell–cell fusion activity as FLv2 ( = FL(I769-V)) (Fig. 5a lane 3, and Fig. 5b panel FL(Q523-L)). Interestingly, when Q523-L and I769-V mutations were separately introduced into S(EP3), both mutants showed a weak cell–cell fusion activity in Vero cells (Fig. 5b panel EP3, EP3(Q523-L) and EP3(I769-V)). The relative cell–cell fusion activities of these S constructs are FL(Q523-L) = FLv2≫EP3(Q523-L)>EP3(I769-V)>EP3. These results reveal that Q523-L and I769-V substitutions are sufficient to compensate the inhibitory effect of F857-L reverse mutation in the FL construct.

### Further enhancement of the cell–cell fusion activity of S protein and adaptation of IBV to cell culture by G405-D substitution

**Figure 6:**
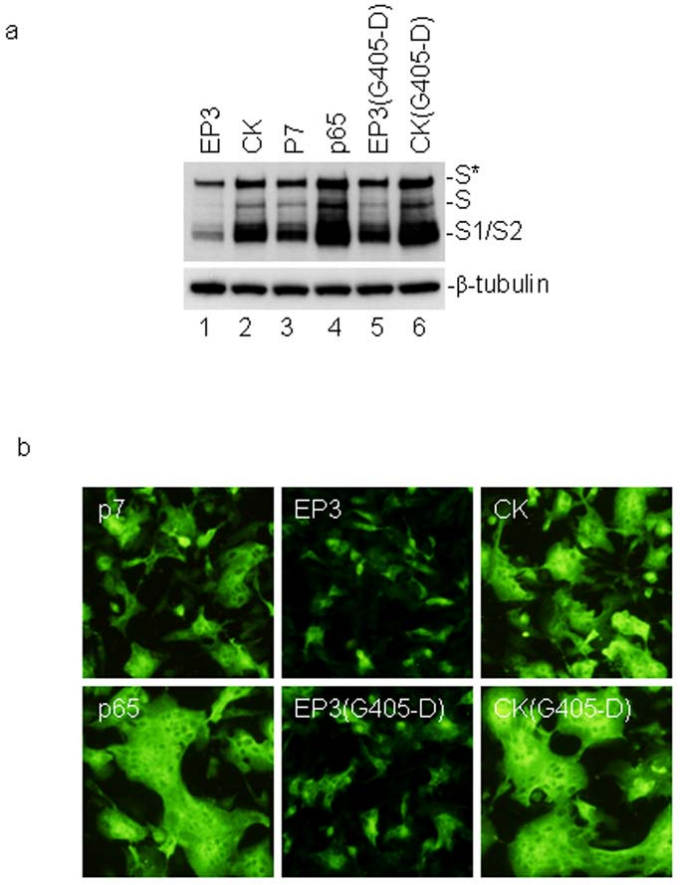
Further enhancement of the cell–cell fusion activity by G405-D mutation in the S1 region. a. Western blot analysis of cells expressing S(EP3), S(CK), S(p7), S(p65), S(EP3(G405D)) and S(CK(G405D)) constructs. Vero cells were infected with vaccinia/T7 recombinant virus and transfected with the indicated constructs. Cells were harvested at 12 hours post-transfection and lysates prepared. The viral protein expression was analyzed by Western blot with rabbit anti-IBV S antibodies. The same membrane was also probed with anti-β-tubulin monoclonal antibody as a loading control. b. Detection of cell–cell fusion by indirect immunofluorescence. Vero cells were infected with vaccinia/T7 recombinant virus and transfected with the indicated S constructs. At 12 hours post-transfecion, cells were fixed with 4% paraformaldehyde and stained with rabbit anti-IBV S polyclonal antibodies.

Vero-adapted IBV gradually increased its infectivity in Vero cells by serial passages and a significant difference between p7 and p65 was observed [10]. Comparison of amino acid sequences between S(p7) and S(p65) revealed a single mutation at the amino acid position 405 (G405-D) in S(p65) (Fig. 1a). To analyze the possibility that the enhanced infectivity of p65 virus is due to the enhanced cell–cell fusion activity of the corresponding S protein, S(p7) and S(p65) constructs were created and expressed in Vero cells. Efficient induction of cell–cell fusion was observed in cells transfected with both constructs (Fig. 6a, panels S(p7) and S(p65) and 6b, lanes 3 and 4). Comparatively, significantly larger syncytia was observed in cells expressing S(p65) construct than in cells expressing S(p7) (Fig. 6a), demonstrating that the additional G405-D mutation in S(p65) may enhance its cell–cell fusion activity. The G405-D mutation was then introduced into S(EP3) and S(CK) and expressed (Fig. 6a), showing that introduction of G405-D mutation into S(CK) drastically enhanced its cell–cell fusion activity (Fig. 6b). Interestingly, introduction of the mutation into S(EP3) and expression of the construct in Vero cells showed formation of small syncytial cells (Fig. 6b). The relative cell–cell fusion activities of these S constructs are CK(G405-D)>p65>CK≫p7>EP3(G405-D)>EP3.

## Discussion

Avian coronaviruses have been isolated from chicken, turkey and pheasant and may exist in many other avian species [20]. IBV is usually associated with respiratory disease in domestic fowl, and was believed to have a limited host range. Chicken is the only natural host. Similarly, coronaviruses originated from human and other animal species were considered to have narrow host specificities until the identification of SARS-CoV as the causative agent of SARS outbreaks in 2003. The current model of animal origin of SARS-CoV highlights the importance of cross-species adaptation and transmission of animal coronaviruses to human. Cross-species transmission of virus infection has long been recognized as a way for the emergence of many zoonotic diseases. The molecular basis for this phenomenon would lie on the rapid adaptation of certain viruses to a changing environment through selection of minor variants from quasispecies, accumulation of mutations, recombination between minor variants, and reassortment of their genomes. A better understanding of the underlying mechanisms that control these events would be essential for providing safeguards to limit the impact of these devastating diseases. In this study, we show that acquisition and enhancement of the cell–cell fusion activity by amino acid substitutions in the S protein are critical for interspecies adaptation and infectivity of IBV to cultured cells. Data present clearly show that the L857-F mutation in the heptad repeat 1 region of S proteins derived from cell-culture-adapted IBV is important for adaptation of the virus to cell culture systems, and an additional mutation in the S1 region (G405-D) could enhance this process. As S protein carrying the L857-F mutation is able to induce cell–cell fusion, but losses the activity when F857 was mutated back to L, it suggests that induction of cell–cell fusion is an essential step in adaptation/selection of IBV to cultured mammalian cells.

Coronavirus S protein is the major determinant for viral entry and host specificity. It is a class I fusion protein and mediates viral entry by specific binding of the S1 domain to a host cell receptor [21]–[25]. The cellular receptors for several coronaviruses have been identified, including members of the cacinoembryonic antigen family of cell adhesion molecules as the receptor for MHV, angiotensin converting enzyme II for SARS-CoV and human coronavirus NL63, and aminopeptidase N for human coronavirus 229E and TGEV [26]–[30]. To date, the receptor(s) for IBV has not been identified in its native or adapted host cells. It is assumed that a mammalian counterpart on Vero cells could be used as a receptor for IBV at low affinity, and might have structural and functional similarities to the native receptor on chicken cells. At the initial stages of the adaptation process, a certain proportion of EP3 would weakly bind to this molecule and gains entry into the cells by endocytosis. In addition, binding of IBV to sialic acid was reported to be important for adaptation of the virus to human cells [31], [32]. The Beaudette strain of IBV was also reported to have an additional binding activity to heparin-like structures [33]. These additional binding activities may help to initiate infection and thus allow the virus to adapt to the new host receptor by mutation.

To uncoat the engulfed virion and to establish subsequent infection cycles as well as to spread infection to neighboring cells, acquisition of virus-cell/cell–cell membrane fusion and enhancement of the cell–cell fusion activity would be an essential step for successful selection/adaptation of virus to the new host cells. Membrane fusion mediated by coronavirus, similar to other viruses, is a multistep process. It includes binding of the S protein to one or more receptors, conformational changes of the protein to a fusion-active form and the actual fusion process. The membrane-fusion activity of coronavirus S protein is mainly associated with domains in the S2 region of the protein [34]–[38]. In this study, we demonstrate that L857-F substitution in the S2 region of the IBV S protein confers the S protein from non-fusogenic to fusogenic. This mutation may affect one of these fusion steps and thus modify the fusion activity of S protein and syncytia formation. At the same time, the virus was successfully adapted to the cultured cells with enhanced infectivity, confirming that acquisition of membrane fusion is an important step in selection/adaptation of IBV to cell culture and may also play a crucial role in cross-species adaptation and transmission of IBV in cultured cells.

Further enhancement of the cell–cell fusion activity of IBV S protein was achieved by a single amino acid substitution (G405-D) in p65 virus. This mutation, meanwhile, enhances the infectivity of the virus in cultured cells. The enhancement effect by mutations in the S1 region and its significance on viral infectivity was further demonstrated by cloning and expression of S gene derived from the IBV variants rescued from the full-length transcripts containing the F857-L mutation. In variants FLv3 and FLv4, additional amino acid substitutions (Q523-L and I769-V) greatly enhanced the cell–cell fusion activity of the L857-containig S protein and the infectivity of the recovered virus. The involvement of residues in the S1 region in the cell–cell fusion activity of S protein was also demonstrated in other coronaviruses [25]. These results illustrate the complexity of the fusion process and the involvement of multiple domains in the induction of membrane fusion. It is worth mentioning that the cell–cell fusion activity of various S constructs was approximated by the degree of cell–cell fusion induced in cells overexpressing individual constructs. In a previous report, we showed nice correlation between the cell–cell fusion activity of two S constructs and their expression levels in the cells [39]. This correlation was also observed in this study with more wild type and mutant S constructs.

**Figure 7:**
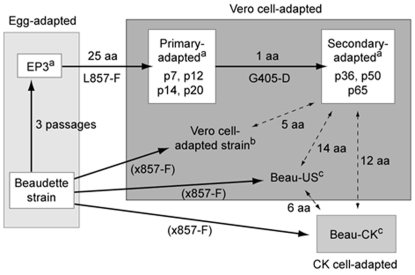
Diagram showing a two-step adaptation process of chicken embryo-adapted IBV to Vero cells. Also shown are the numbers of amino acid changes during each adaptation process. ^a^The accession No. for S genes from EP3 is DQ001338, p7 is DQ001337, p65 is DQ001339. ^b^The accession No. for this Vero-adapted strain is AAV98206. ^c^The accession No. for these two strains are CAC39114 and CAC39300.

Based on data generated from adaptation [10] and cell–cell fusion studies presented here, a model of two-step adaptation process is proposed (Fig. 7). In this model, the adaptation was divided into primary and secondary adaptation (Fig. 7). Early passages of Vero-adapted IBV, including p7, p12, p14 and p20, belong to the primarily adapted group (Fig. 7). Other cell culture-adapted strains, including the CK-adapted and Beaudette-US strains CAC39114 and CAC39300 [8], [9], and the Vero-adapted strain AAV98206 described by Youn et al. [13], also belong to this group (Fig. 7). Late passages of Vero-adapted IBV, including p36, p50 and p65, contain the additional G405-D amino acid substitution and belong to the secondarily adapted group (Fig. 7).

Except in the cell culture-adapted IBV strains, the L857 residue was found to be absolutely conserved in all coronaviruses sequenced so far. As structural information for this IBV S protein is currently lacking [40], [41], we are unclear the overall role of this residue on the formation and stability of the six-bundle structure of the protein. Further structural and functional studies are required to delineate the precise roles of this mutation in the fusion process. Mutation of this residue to either a Ser or a Tyr showed similar effect on the cell–cell fusion activity of the S protein as a Phe. On the other hand, when the residue was mutated to Ile, Glu or Lys, a much lower cell–cell fusion activity of the S protein was observed. Interestingly, mutations in the S1 and some distant S2 regions could compensate the effect of F857-L mutation. This may explain why S protein from several other coronaviruses, such as MHV and human coronavirus, could induce efficient virus-cell/cell–cell fusion although a conserved Leu residue was found at the equivalent position [42], [43].

It is worth mentioning that the cell–cell fusion activities of different S constructs were qualitatively and semi-quantitatively determined in cells overexpressing individual S constructs using the vaccinia/T7 expression system. Attempts to obtain more rigorous quantitative data were unsuccessful. As shown in this study, S protein is inefficiently translocated to the cell surface, probably due to the presence of an ER retention signal [44]. Since cell surface expression of S protein is a prerequisite for the induction of cell–cell fusion, disruption of the ER-retention signal may facilitate cell surface expression as well as quantitative analysis of the cell–cell fusion activity of the protein.

As virus-cell/cell–cell fusion is essential for efficient propagation of viral infection, attempts to interfere this process with fusion inhibitors, such as peptides or small molecules, are being made for several viruses, including HIV, SARS-CoV and influenza virus. The involvement of multiple domains in the induction of cell–cell fusion demonstrated here would complicate the design of such inhibitors. Furthermore, mutations in regions beyond the target sequence, in the case of coronaviruses the S1 and some distant S2 regions, may lead to the emergence of drug-resistant strains. Understanding of the fusion mechanisms in more detail would, therefore, help design more efficient inhibitors.
